# Magnetic resonance imaging and magnetic resonance venography features in heat stroke: a case report

**DOI:** 10.1186/s12883-019-1363-x

**Published:** 2019-06-18

**Authors:** Lizhi Cao, Juan Wang, Yaxuan Gao, Yumei Liang, Jinhua Yan, Yunhai Zhang, Mingqin Zhu, Tianfei Luo, Jiafeng Chen

**Affiliations:** 1 Department of Neurology, The First Hospital of Jilin University, Changchun, 130021 Jilin China; 2 Norman Bethune Health Science Center of Jilin University, Changchun, 130000 Jilin China; 3 Jiangsu Key Laboratory of Medical Optics, Suzhou Institute of Biomedical Engineering and Technology, Chinese Academy of Sciences, Suzhou, 215163 China

**Keywords:** Heat stroke, Cerebral venous thrombosis, Magnetic resonance imaging, Magnetic resonance venography, Brain lesions

## Abstract

**Background:**

Heat stroke (HS) is a critical illness that can cause multiple organ dysfunction, including damage to the central nervous system (CNS), which can be life-threatening in severe cases. Brain lesions in patients with HS who present with CNS damage have been rarely reported before, and they usually vary in different cases, hence, patients with such lesions may present a clinical challenge in terms of diagnosis and management. Cerebral venous thrombosis (CVT) is a rare cause of stroke that mostly affects young individuals and children. The pathogenesis of brain damage caused by HS is complex, and CVT may be involved in the pathogenesis of HS with CNS damage. In this manuscript, we have reported a case of a patient with HS having CVT with symmetrical lesions in the bilateral putamen, posterior limb of the internal capsule, external capsule, insular lobe, and subcortical white matter in the brain.

**Case presentation:**

We encountered a 48-year-old man who presented with HS in the summer season. During admission, he had a high body temperature and was in coma and shock. Then, he developed rhabdomyolysis syndrome, acute kidney and liver damage, electrolyte imbalance, and acid–base balance disorders, and his D-dimer level was elevated. After several days of anti-shock treatment, the patient’s level of consciousness improved. However, he experienced a decline in vision. Cerebral magnetic resonance imaging (MRI) showed symmetrical lesions in the bilateral posterior limb of the internal capsule, putamen, external capsule, insula, and subcortical white matter, and cerebral magnetic resonance venography (MRV) showed the development of CVT. Therefore, anti-coagulation treatment was provided. After timely clinical intervention, the symptoms of the patient gradually improved.

**Conclusions:**

This case showed that HS can cause CVT. Therefore, cerebral MRI findings in HS must be assessed; in addition, early MRV can help in the diagnosis of the disease, which can effectively improve prognosis.

## Background

Heat stroke (HS) is a systemic inflammatory response syndrome with a pathophysiological process similar to that of severe sepsis. The main clinical symptoms of such a condition are core body temperature greater than 40 °C and multiple organ dysfunction, including damage to the central nervous system (CNS). CNS abnormalities include inattention, memory loss, paralysis, convulsions, and coma. Multiple organ dysfunction syndrome can occur in critically ill patients. The common complications of HS are acute respiratory distress syndrome, disseminated intravascular coagulation (DIC), shock, rhabdomyolysis, acid–base or electrolyte disorders, renal failure, cerebral edema, and liver dysfunction [1, 2]. Changes in the cerebral image of patients with HS have been rarely reported, and the mechanism of CNS damage caused by HS is not fully understood. In addition, it remains unclear which parts of the brain are more susceptible to damage caused by HS.

Cerebral venous thrombosis (CVT) is a rare form of cerebrovascular disease. Young individuals and children constitute the main patient groups with CVT. Due to the age of onset and the different causes of CVT, its clinical manifestations are diverse. The common clinical manifestations of CVT include high intracranial pressure symptoms (headache, papilledema, and vomiting), focal symptoms, and encephalopathy-like symptoms. Encephalopathy-like symptoms are rare; however, most symptoms are severe, and the patient can develop epilepsy, mental disorders, and confusion and can go into coma [3]. CVT is associated with several risk factors, which include pregnancy/puerperium, use of oral contraceptive, dehydration, cancer, and infections. The most common brain parenchymal lesions of CVT are intracerebral infarction and hemorrhage [4], followed by focal cerebral edema, such as lesions in the thalamus and basal ganglia, caused by obstruction in the deep venous system [5].

In this article, we report a case of HS with CVT with symmetrical lesions in both sides of the basal ganglia.

## Case presentation

**Fig. 1:**
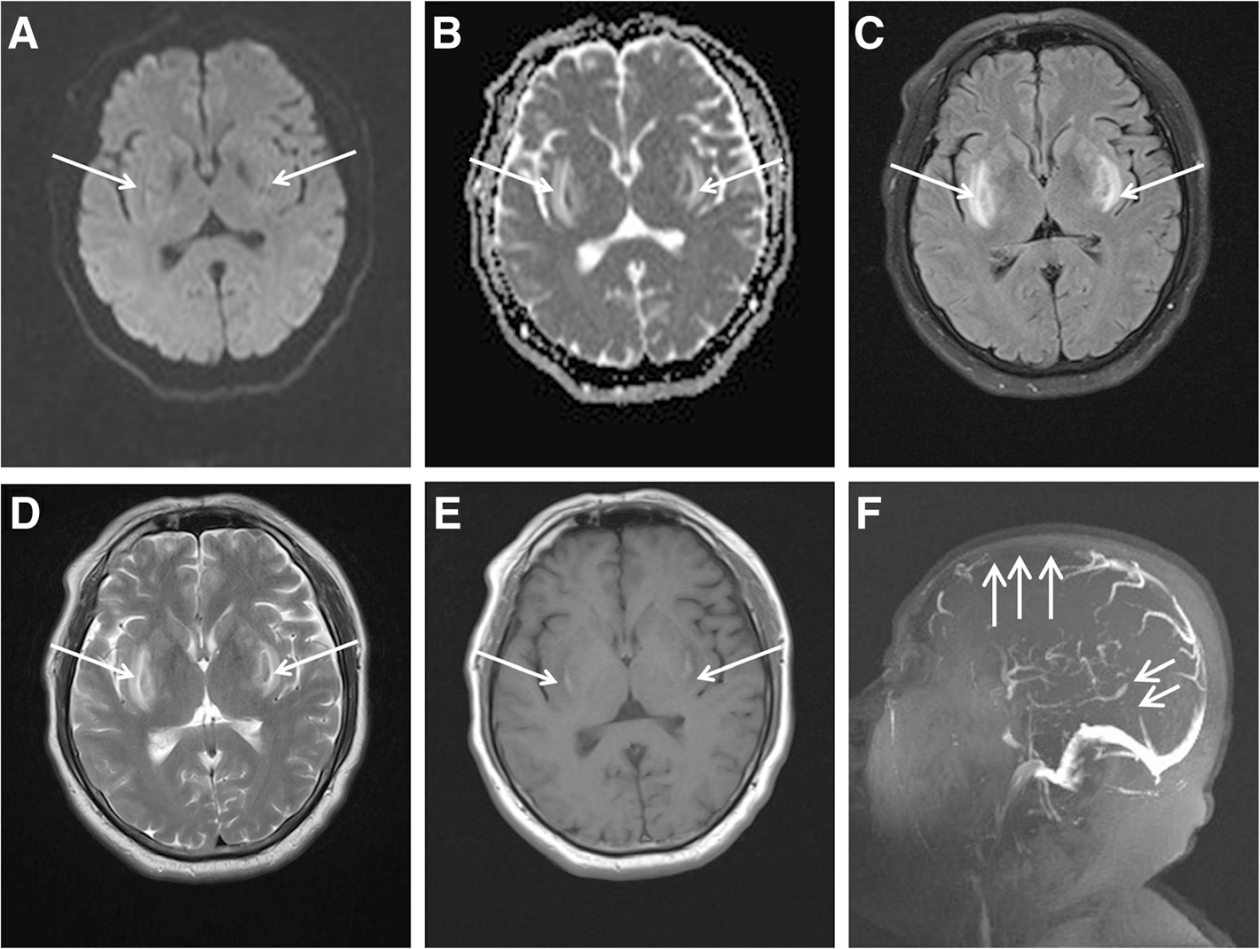
**a**-**f.** Brain magnetic resonance imaging (MRI) was performed 8 days after admission. Cerebral MRI revealed a slight hyperintense signal in the bilateral putamen on **a** diffusion-weighted imaging sequence and bilateral symmetrical hypointensity in the middle of the putamen as well as hyperintensity around hypointensity on **b** apparent diffusion coefficient, **c** fluid-attenuated inversion recovery, and **d** T2-weighted imaging sequence. The lesions were hyperintense in the middle of the bilateral putamen and hypointense around them on **e** T1-weighted imaging sequence. **f** The inferior sagittal sinus, straight sinus, and vein of Galen were not observed on magnetic resonance venography (MRV) performed on the 12th day of admission. The superior sagittal sinus was poorly developed based on MRV

**Fig. 2:**
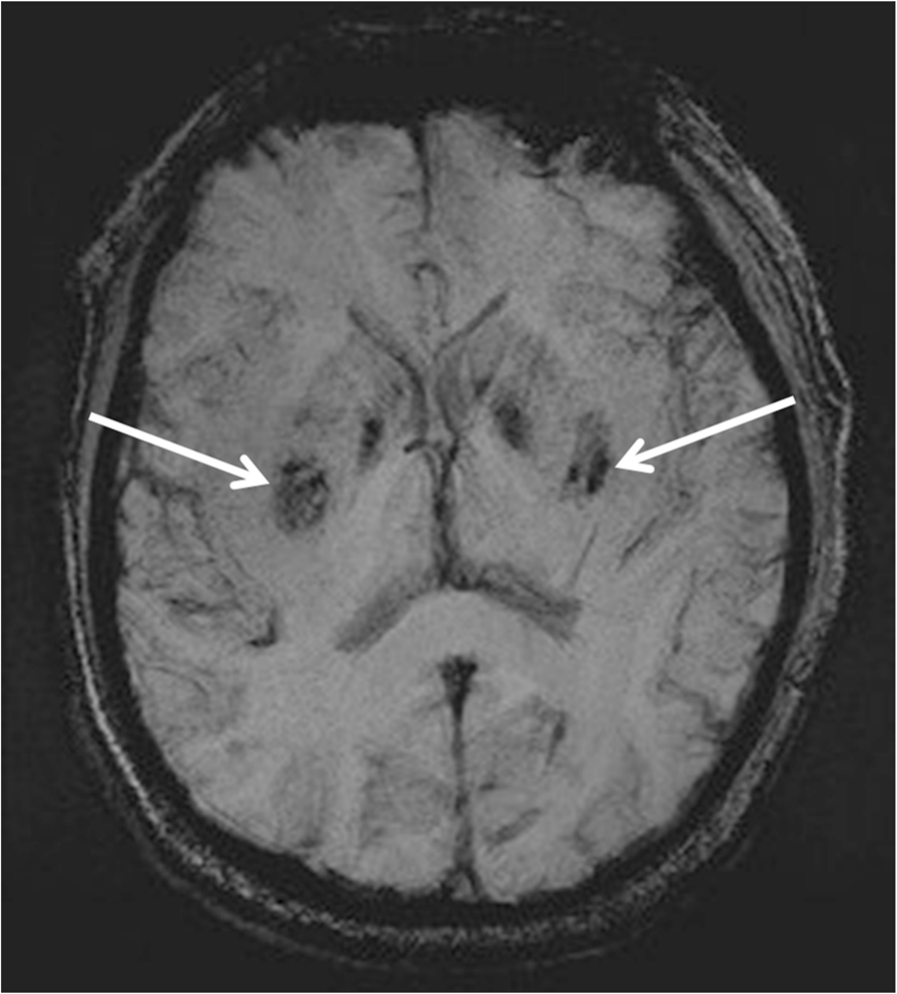
Susceptibility-weighted imaging conducted on day 17 indicated bilateral hemosiderin deposition or hemorrhagic foci in the basal ganglia

**Fig. 3:**
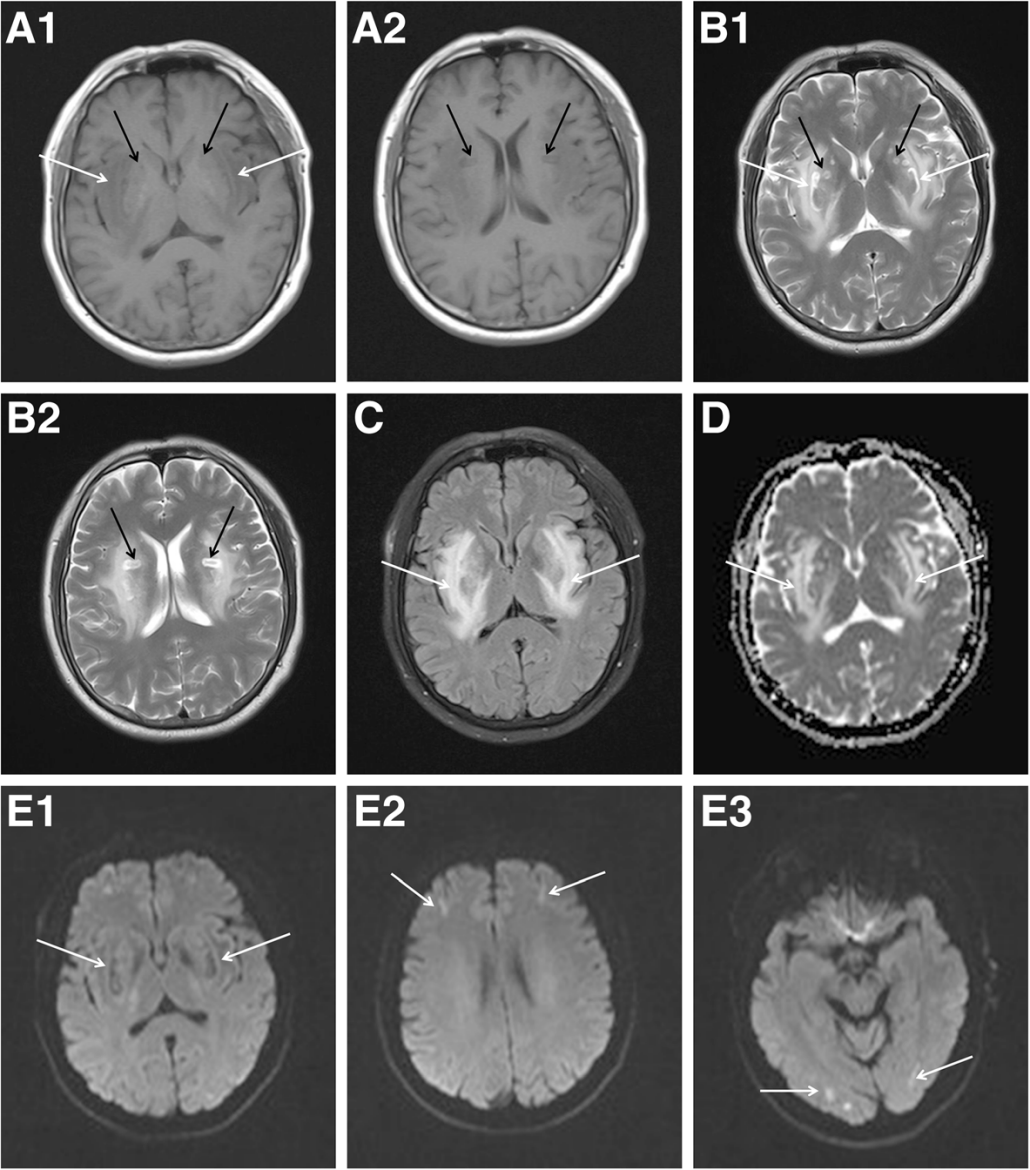
**a**1–**e**3. Follow-up magnetic resonance imaging obtained 25 days after admission showed symmetrical abnormal signals in the bilateral posterior limb of the internal capsule, putamen, extern al capsule, and insular lobe. The signals were hypointense on T1-weighted imaging sequence (T1WI) **(a1,** white arrows**)** and hyperintense on T2-weighted imaging sequence (T2WI) **(b1,** white arrows**)**, **(c)** fluid-attenuated inversion recovery, and **(d)** apparent diffusion coefficient . They were not limited by diffusion on **(e1)** diffusion-weighted imaging (DWI). Strip and dot-like signals, which were isointense and slightly hypointense on T1WI **(a1–2,** black arrows**)** and hypointense on T2WI **(b1–2,** black arrows**)**, can be observed in the lesions. **(e2–3)** DWI revealed bilateral hyperintensity in the frontal and occipital lobes

**Fig. 4:**
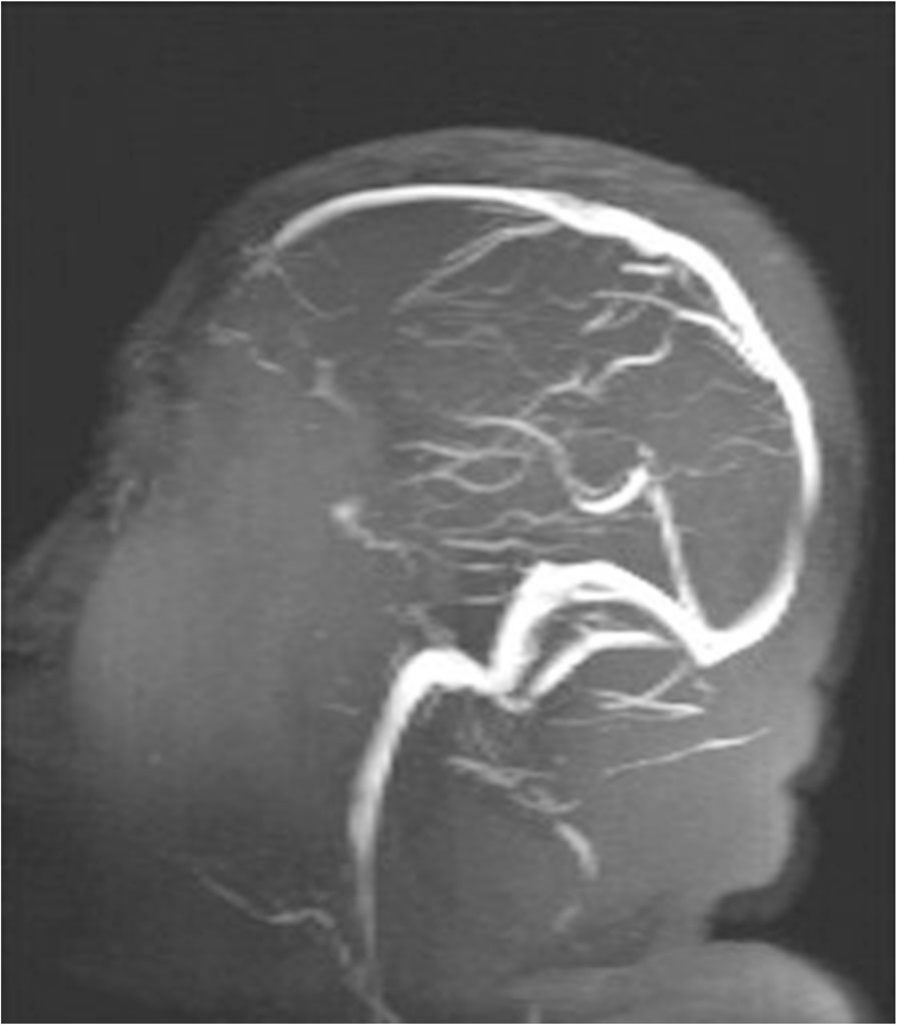
The flow signals of the superior sagittal sinus, straight sinus, and vein of Galen were significantly better on follow-up magnetic resonance venography performed on the 25th day after admission

**Fig. 5:**
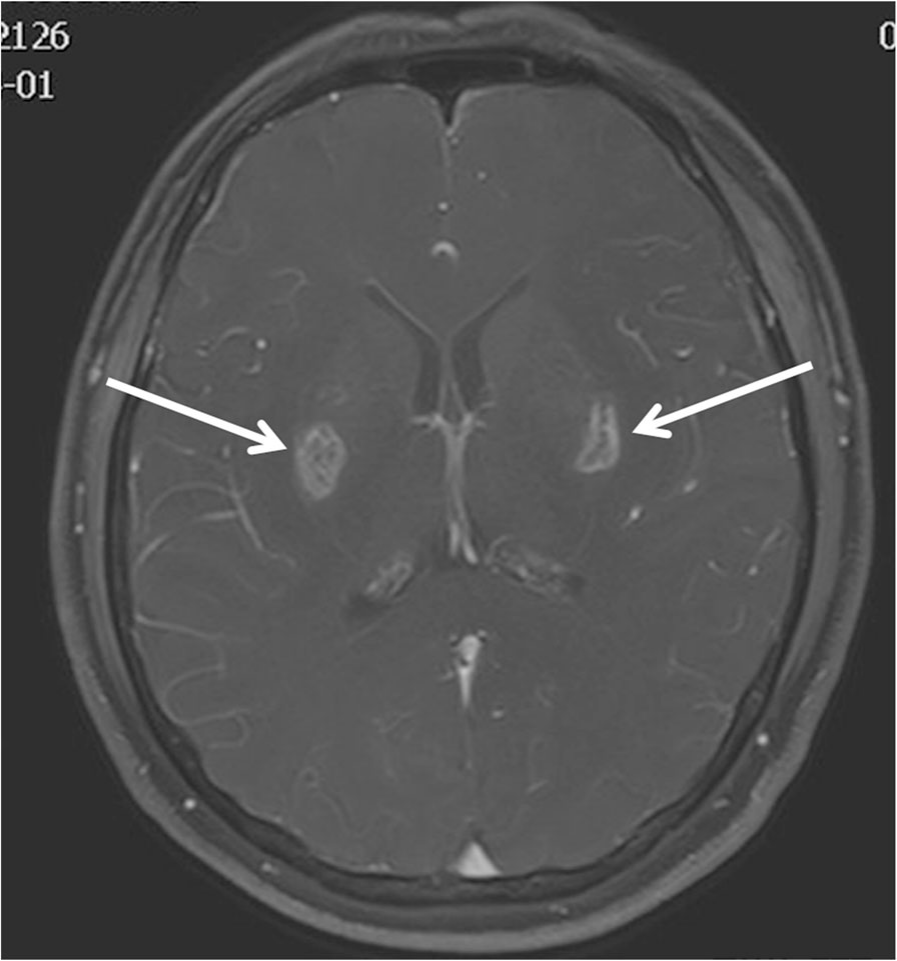
On day 28, magnetic resonance imaging (MRI) after gadolinium was administered revealed abnormal enhancement within the bilateral basal ganglia, and the size of the lesions decreased on MRI conducted 25 days after admission

During a hot afternoon in July, a 48-year-old man developed symptoms, such as nausea, vomiting, headache, chest tightness, and shortness of breath, while working outdoors for 2 h in a southern city in China. The outside temperature at that time was 35 °C. Notably, the patient lived in the north part of China and had traveled to the southern part when he was sick. After 15 h, he went into coma and was then transferred to the emergency department of the hospital. The patient was in good health before the onset of the disease. Upon arrival, the temperature of the patient was 40.2 °C, blood pressure was 75/40 mmHg, and pulse rate was 100 beats/min. His blood oxygen saturation under balloon-assisted ventilation was 95%. Laboratory tests indicated rhabdomyolysis syndrome, acute kidney injury, hepatic disfunction, hyperkalemia, and metabolic acidosis. The serum D-dimer level of the patient was elevated at 1022 (normal range: 0–232) μg/L. Therefore, the patient was diagnosed with HS. He was immediately treated with a cooling blanket and plasma exchange and received assisted ventilation. Brain CT scans performed on the 3rd day of admission showed symmetrical low-density lesions in the bilateral basal ganglia. On the 7th day of admission, the patient’s state of consciousness improved. However, he experienced blurred vision. Eye examination results were normal. Brain magnetic resonance imaging (MRI) was performed 8 days after admission. Cerebral MRI revealed a slight hyperintensity in the bilateral putamen on diffusion-weighted imaging (DWI) sequence and bilateral symmetrical hypointensity in the middle of the putamen and hyperintensity around hypointensity on the apparent diffusion coefficient (ADC), fluid-attenuated inversion recovery (FLAIR), and T2-weighted imaging (T2WI) sequence. The lesions showed hyperintensity in the middle of the bilateral putamen and hypointensity around them on T1-weighted imaging (T1WI) sequence (Fig. 1A-E). Magnetic resonance venography (MRV) in sagittal projection performed on the 12th day of admission showed the absence of a straight sinus and vein of Galen, indicating CVT. In addition, the lack of flow signal was also found in the distal part of the superior sagittal sinus that also corresponds to CVT (Fig. 1F). Intravenous treatment of mannitol, subcutaneous injection of low-molecular-weight heparin calcium (5000 IU, two times/day) was initiated to reduce high intracranial pressure and to treat CVT. Cerebrospinal fluid (CSF) examination conducted on day 17 showed elevated protein levels at 1.87 (normal range: 0.15–0.45 g/L and immunoglobulin G levels at 267.0 (normal range: 0–34.0) mg/L. The CSF pressure was 210 (normal range: 80–180) mmH_2_O. Susceptibility-weighted imaging (SWI) obtained on the same day indicated bilateral hemosiderin deposition or hemorrhagic foci in the basal ganglia (Fig. 2). Follow-up MRI obtained 25 days after admission showed symmetrical abnormal signals in the bilateral posterior limb of the internal capsule, putamen, external capsule, and insular lobe. The signals were hypointense on T1WI and hyperintense on T2WI, FLAIR, and ADC and were not limited by diffusion on DWI. Strip and dot-like signals, which were isointense and slightly hypointense on T1WI and hypointense on T2WI, can be observed in the lesions. DWI revealed bilateral hyperintensity on the frontal and occipital lobes (Fig. 3A1–E3). The flow signals of the superior sagittal sinus, straight sinus, and vein of Galen were significantly better on follow-up MRV (Fig. 4). On the 28th day of admission, after the administration of gadolinium, MRI revealed abnormal enhancement within the bilateral basal ganglia, and the size of the lesions decreased on MRI conducted 25 days after admission (Fig. 5). The patient was discharged with blurred vision on the 38th day. (Timeline of brain imaging was shown in Table 1).

**Table 1:** Timeline of Brain Imaging

Day	Brain CT scan/MRI Findings	Brain MRV Findings
3	Symmetrical low-density lesions in the bilateral basal ganglia on CT scan.	ND
8	Slight hyperintensity in the bilateral putamen on DWI. Bilateral symmetrical hypointensity in the middle of the putamen and hyperintensity around hypointensity on ADC, FLAIR, and T2WI. Hyperintensity in the middle of the bilateral putamen and hypointensity around them on T1WI.	ND
12	ND	Inferior sagittal sinus, straight sinus, and vein of Galen were not observed on MRV. The superior sagittal sinus was poorly developed on MRV.
17	Bilateral hemosiderin deposition or hemorrhagic foci in the basal ganglia on SWI.	ND
25	Symmetrical abnormal signals in the bilateral posterior limb of the internal capsule, putamen, external capsule, and insular lobe. The signals were hypointense on T1WI and hyperintense on T2WI, FLAIR, and ADC and were not limited by diffusion on DWI. Strip and dot-like signals, which were isointense and slightly hypointense on T1WI and hypointense on T2WI, can be observed in the lesions. Bilateral hyperintensity on the frontal and occipital lobes on DWI.	The flow signals of the superior sagittal sinus, straight sinus, and vein of Galen were significantly better on follow-up MRV.
28	Abnormal enhancement within the bilateral basal ganglia on MRI after the administration of gadolinium.	ND

“ND” represents “Not Done”

## Discussion and conclusions

Severe HS can be life-threatening, and nearly 30% of survivors present with permanent neurological sequelae [1]. The damage in the CNS due to HS is caused by a variety of factors. Heat itself is toxic to the brain cells (such as Purkinje cells of the cerebellum) [6]. Excessive secretion of cytokines, such as interleukin-1, can disrupt the blood–brain barrier, which in turn leads to vasogenic edema [7]. Lesions in a patient with HS that are hyperintense on DWI and hypointense on ADC may indicate cell-derived edema rather than vasogenic edema [8]. DIC can cause intracerebral hemorrhage, and microthrombus derived from DIC can cause small vessel ischemic injury. Incomplete circulatory function can lead to cerebral ischemia and hypoxic injury. Myelin can dissolve due to metabolic disorders [9]. In addition, recent studies have shown that excitotoxic injury may also be involved in the pathogenesis of HS. Li J and his coworkers have found that the N-acetyl aspartate/creatine value was low on magnetic resonance spectroscopy imaging of patients with HS [10].

For several years, only few cases with imaging findings of the CNS have been reported. Brain lesions caused by HS are usually symmetrical on imaging examination. Based on MRI findings in the literature, the lesions are mainly distributed in the cerebellum, thalamus, basal ganglia, cerebral cortex, brainstem, hippocampus, subcortical white matter, external capsule, and splenium. The lesions of the cerebellum are mainly concentrated in the cerebellar cortex, superior cerebellar peduncle, vermis of the cerebellum, and corpora dentatum. In addition, the common lesion site is the caudate nucleus of the basal ganglia [6, 7, 9, 11–18]. However, our case involved symmetrical lesions in both sides of the posterior limb of the internal capsule, putamen, external capsule, insular lobe, and subcortical white matter, which has not been reported before. Due to the selective vulnerability of cerebellar neurons and Purkinje cells to thermal damage [14, 19], the cerebellum is likely to be damaged in HS. Several patients with HS present with cerebellar symptoms, such as ataxia [20]. Although some studies have found cerebellar atrophy delays on radiographic images [21], no abnormal signals were found in the cerebellum in our patient on MRI.

The literature shows that brain damage caused by HS appears as hyperintense lesions on the following imaging sequences: T2WI and FLAIR [9, 11], DWI and FLAIR [12], and T1WI and T2WI. Sometimes these anomalous signals are limited to DWI [13]. However, the lesions in our case showed high and low mixed signals in the abovementioned sequences. Lesions can be enhanced occasionally on contrast-enhanced imaging. Cerebral lesions in patients with HS showed punctiform hemorrhage on SWI [22].

Shock is one of the numerous risk factors of CVT [23]. Dentali F et al. have reported that D-dimer in CVT has a sensitivity of 94% [24]. The deep venous system consists of the straight sinus, vein of Galen, and internal cerebral veins. MRI of deep cerebral venous thrombosis (DCVT) can often observe bilateral thalamic lesions involving the basal ganglia [25]. During admission, the patient was in shock and his D-dimer level was elevated. Due to the presence of bilateral basal ganglia lesions, we prescribed MRV examination and found some abnormalities. In the literature, data about MRV changes in patients with HS are limited; thus, the findings in this case must be reported. Interestingly, the patient’s lesions were mainly located in the bilateral basal ganglia, not in the thalamus, which is different from the common lesions of DCVT. Two previous studies have shown that low-molecular-weight heparin is more suitable for the treatment of uncomplicated CVT than unfractionated heparin [26, 27]. In general, patients with CVT can achieve a good prognosis after treatment [28]. In a study by Arauz A et al., approximately 90% of patients with obstructed cerebral veins were recanalized [29]. In our study, after the patient was treated with low-molecular-weight heparin for 13 days, the second MRV was performed on the 25th day of admission, and results showed recanalization of the obstructed cerebral veins. On day 38, the patient was discharged with blurred vision, and the Modified Rankin Scale score was 2.

The mechanism of brain damage caused by HS is complex and diverse, and its cerebral imaging changes vary. Herein, we have reported a patient with high and low mixed signals in the bilateral posterior limb of the internal capsule, putamen, external capsule, insula, and subcortical white matter on MRI, and his MRV showed development of CVT. After treatment, the obstructed cerebral veins were recanalized. We believe that when the cerebral MRI findings of patients with HS need to be identified, MRV and other related tests should be performed. Moreover, timely treatment can improve prognosis.

## Data Availability

All data generated or analysed during this study are included in this published article.

## Abbreviations

ADC: Apparent diffusion coefficient
CNS: Central nervous system
CSF: cerebrospinal fluid
CVT: Cerebral venous thrombosis
DCVT: Deep cerebral venous thrombosis
DIC: Disseminated intravascular coagulation
DWI: Diffusion-weighted imaging
FLAIR: Fluid-attenuated inversion recovery
HS: Heat strok
MRI: Magnetic resonance imaging
MRV: Magnetic resonance venography
SWI: Susceptibility-weighted imaging
T1WI: T1-weighted imaging
T2WI: T2-weighted imaging
